# Triterpenoids – future multi-target compounds against obesity and towards restoration of metabolic health

**DOI:** 10.3389/fragi.2026.1939566

**Published:** 2026-10-27

**Authors:** Johanna Becker, Birgitta E. Ebert, Gunter Festel, Lars M. Blank

**Affiliations:** 1 iAMB – Institute of Applied Microbiology, ABBt – Aachen Biology and Biotechnology, RWTH Aachen University, Aachen, Germany; 2 School of Chemistry and Molecular Biosciences, The University of Queensland, Brisbane, QLD, Australia; 3 Swiss Institute of Longevity, Fuerigen, Switzerland; 4 TriTerp, Fuerigen, Switzerland

**Keywords:** autophagy, longevity, metabolic health, obesity, triterpenoids

## Abstract

Obesity and its associated diseases represent one of the most urgent public health crises of our time, affecting billions worldwide. Current pharmacological agents, such as GLP-1 and GLP-1/GIP receptor agonists, have demonstrated high clinical efficacy in diabetes and weight management. However, the required lifelong medication is often limited by high economic costs and severe side effects, including gastrointestinal complications. Consequently, the search for complementary therapies is invaluable. This review highlights the potential of natural triterpenoids in obesity management by shifting the focus towards long-term metabolic restoration. Triterpenoids can serve as multi-target agents that address key pathways, such as AMPK and Akt/mTOR, thereby positively regulating autophagy processes and improving metabolic imbalances. Celastrol, for example, acts as a leptin sensitizer, restoring satiety signaling; ursolic acid drives body recomposition by enhancing energy expenditure; and ginsenosides modulate multiple processes, including browning, adipogenesis, lipogenesis, and satiety signaling. Furthermore, oleanolic acid promotes pathways associated with cellular healthspan by activating telomerase, while betulinic acid suppresses *de novo* fatty acid synthesis independently of food intake. However, low oral bioavailability remains the major challenge to successfully translating these findings from animal models to clinical applications. Advanced strategies, such as sustainable bioproduction via genetically modified microorganisms, engineered probiotics, or nanotechnology-based delivery systems, offer promising opportunities to overcome these pharmacokinetic and safety limitations. Addressing these barriers is essential to translate triterpenoids into therapeutic agents for human metabolic health and healthy aging.

## 1 Introduction

Due to increasing life expectancy, the study of healthy aging is gaining unprecedented importance–focusing not only on extending human lifespan, but primarily on preserving healthspan and shortening periods of morbidity in old age (Festel, 2024). However, healthy aging requires preventing and treating chronic metabolic diseases.

In this context, obesity has emerged as a global epidemic and one of the most critical public health challenges of our time. It is characterized by excessive fat accumulation in the body and is commonly defined by body mass index (BMI), a surrogate marker of adiposity. The BMI is calculated by dividing an individual’s weight in kilograms by the square of their height in meters [kg/m^2^]. For adults, a BMI of 25.0–29.9 kg/m^2^ is classified as overweight, while a BMI of 30.0 kg/m^2^ or higher is categorized as obese (NCD Risk Factor Collaboration, 2024).

In 2022, 2.5 billion adults worldwide were graded as overweight, including 890 million adults (representing 16% of the global adult population) living with obesity (World Health Organization, 2025). Current trends suggest that by 2030, approximately 50% of the global adult population will be overweight (BMI ≥ 25.0), with 17% of men and 22% of women expected to have obesity (World Obesity Federation, 2025). This alarming trend spans young people (5–19 years) as well: the number of overweight or obese youth rose from 143 million in 2015 to over 390 million in 2022, totaling 20% of the global youth population. By 2030, an estimated 464 million children and adolescents will be overweight or obese (World Health Organization, 2025).

Obesity results from an imbalance in energy metabolism. The causes driving this dramatic surge are diverse: genetic predispositions; epigenetic factors such as sedentary lifestyles, the wide availability of sugar-sweetened beverages, and the rising consumption of nutrient-poor and energy-dense food; and hormonal imbalances, including growth hormone deficiency, leptin resistance, and Cushing’s syndrome (Bouchard, 2021; Mahmoud, 2022; Masood and Moorthy, 2023; World Health Organization, 2025).

The rising prevalence of obesity has led to increasing health concerns. In 1997, the World Health Organization declared obesity a global epidemic, establishing it not as a lifestyle choice but as a chronic disease (Bouchard, 2021). It was identified as a major contributor to ill health, with extensive consequences. This classification is based on its association with severe comorbidities such as type 2 diabetes, hypertension, coronary heart disease, and certain forms of cancer, all of which significantly impair both quality of life and longevity (Kopelman, 2000; Lu and Li, 2000). In 2021, overweight and obesity caused approximately 3.7 million deaths from noncommunicable diseases such as diabetes or cancer (World Health Organization, 2025). This growing burden underscores the urgent need for effective therapeutic strategies.

Current obesity treatment focuses on established pharmacotherapies, including the use of glucagon-like peptide-1 (GLP-1) and GLP-1/glucose-dependent insulinotropic polypeptide (GIP) receptor agonists (e.g., Semaglutide in Wegovy® and Ozempic®, Tirzepatide in Mounjaro® and Zepbound®), which have demonstrated significant efficacy in weight loss and metabolic benefits (Zhu et al., 2024; Berning et al., 2025). However, the required lifelong medication is often limited by high economic costs and severe adverse effects, including gastrointestinal problems, potential micronutrient deficiencies, and dehydration (Cuschieri et al., 2025). Consequently, complementary strategies are invaluable, such as exploring natural bioactive compounds that modulate metabolic pathways.

Natural triterpenoids, a diverse class of secondary metabolites found in plants, fungi, and sea cucumbers, have emerged as promising candidates to shift from simple weight management to long-term metabolic restoration, thereby promoting metabolic health and cellular mechanisms of healthy aging. In this context, metabolic restoration is defined as the body’s return to its original metabolic homeostasis. This process includes multiple physiological improvements, such as reduced lipid levels, reversal of insulin and leptin resistance, reduced pro-inflammatory cytokines including interleukin-6 and tumor necrosis factor-α, and normalized blood glucose levels (Shahnawaz and Sharma, 2026).

The application of multi-target compounds, and the subsequent simultaneous modulation of multiple metabolic mechanisms, is highly advantageous for the effective treatment of multifactorial diseases like cancer, diabetes, and obesity (polypharmacology), as addressing single targets in the disease network is often insufficient to achieve optimal therapeutic benefits and restore the system to a healthy homeostatic state (Zimmermann et al., 2007). However, it must be mentioned that this multi-target activity also carries an inherent risk of off-target interactions (promiscuity), leading to narrow therapeutic windows, agent-specific toxicities, and adverse effects. Therefore, multi-target therapeutics require a meticulous balance between therapeutic efficacy and safety compared with single-targeted agents.

The following sections address specific triterpenoids (celastrol, ursolic acid, ginsenosides, oleanolic acid, and betulinic acid) selected based on criteria including their relevance to obesity research, polypharmacology, and potential for clinical translation. However, a critical translation gap persists, as promising effects in rodent models often translate into no significant or only modest, inconsistent reductions in human body weight. This discrepancy largely stems from low oral bioavailability and cytotoxicity, the key bottlenecks for triterpenoid clinical applications. We briefly mention additional triterpenoids and their derivatives (mogrosides, boswellic acids, cucurbitacins, *Centella* triterpene saponins) because of their potential as emerging compounds. Table 1 provides a quantitative overview of the selected primary triterpenoids, including study details such as model, dosage, duration, primary outcomes, and key molecular and tissue alterations induced. The main text critically discusses emerging compounds; this table excludes them because most studies report extract-level findings, and few provide compound-specific evaluations.

**TABLE 1:** Quantitative overview of selected primary triterpenoids, including study details such as model, dosage, duration, primary outcomes, and key molecular and tissue alterations induced. Emerging compounds (e.g., boswellic acids, mogrosides, cucurbitacins, and *Centella* triterpene saponins) are critically discussed in the main text and excluded from this quantitative table due to frequent investigations at the extract level and a current deficiency of compound-specific evaluations.

Triterpenoid	Model	Dosage	Duration	Primary outcomes	Key molecular and tissue alterations	REFs
Celastrol	DIO C57BL/6J mice	100 μg/kg/day	3 weeks	45% weight loss; ∼75% less food intake	sensitizer of hypothalamic leptin via PTP1B and TCPTP inhibition	(Liu et al., 2015)
Ursolic acid	DIO C57BL/6 mice	0.14%–0.27% (w/w)	6 weeks	significant reduction of total body weight; decrease in fasting blood glucose levels and liver weight	significant increase in skeletal muscle (quadriceps and triceps weight) and brown adipose tissue mass via stimulation of IGF1/Akt pathway	(Kunkel et al., 2012)
Ginsenosides (Rb1, Rg1, Rg3, and Compound K)	Rb1: 3T3-L1 preadipocytes and primary white adipocytes Rg1: DIO Sprague-Dawley rats Rg3: DIO C57BL/6J mice CK: 3T3-L1 preadipocytes	Rb1: 10, 20, and 40 μM Rg1: 25 and 100 mg/kg/day Rg3: 10 mg/kg CK: 0–40 μM	Rb1: 7 days Rg1: 5 weeks Rg3: 12 weeks CK: 8 days	reduction in body weight and tissue adiposity; decrease in fasting blood glucose, TAG, and total cholesterol levels	all: activation of AMPK phosphorylation, driving the downstream suppression of lipogenesis and adipogenesis genes (C/EBPα, SREBP-1c) Rb1, Rg1, and Rg3: upregulated expression of PPARγ, UCP1 and PGC-1α induces WAT browning Rg1 specific: promotion of Nrf2 pathways and downregulation of Aldh3a1, Aox3, and Dct to reduce hypothalamic oxidative stress Rg3 specific: stimulation of GLP-1 secretion, promotion of satiety signaling CK specific: suppression of ERK, p38, and Akt phosphorylation, driving the inhibition of preadipocyte differentiation	(Park H et al., 2019; Pu et al., 2022) (Huang et al., 2026) (Kim et al., 2015; Mu et al., 2021) (Oh and Chun, 2022)
Oleanolic acid	HepG2 cells DIO C57BL/6J mice DIO C57BL/6 mice human peripheral blood mononuclear cells	100 + 150 μM 30, 60 + 120 mg/kg/day 50 mg/kg/week 1 μg/mL	24 h 8 weeks 6 months 24–72 h	reduced levels of total cholesterol and triglycerides; improved liver steatosis; reduced adipogenesis; no toxic effects with c_OA_ ≤ 100 μM reduced weight gain; enhanced skeletal muscle mass 5.9-fold enhanced telomerase activity in comparison to untreated control cells	reduces expression levels of PPARγ, C/EBPα, C/EBPβ, SREBP-1c; attenuated levels of inflammatory factors regulation of PI3K/Akt signaling upregulation of telomerase function	(Claro-Cala et al., 2022; Zhang et al., 2024) (Sun et al., 2024b) (Tsoukalas et al., 2019)
Betulinic acid	DIO C57BL/6 mice DIO Swiss mice	150 mg/kg/day 50 mg/L in drinking water (∼40 mL/week net water intake)	11 weeks 15 weeks	decreased body weight (∼13%), abdominal fat accumulation (∼1.6-fold), lipid droplet size, blood glucose, TAG, and cholesterol levels (∼24%); insulin sensitizer	upstream activation of AMPK, driving the suppression of lipogenesis- and adipogenesis-related gene expression	(Kim et al., 2019) (de Melo et al., 2009)

Abbreviations: Aldh3a1 – aldehyde dehydrogenase 3 family member A1; Akt–protein kinase B; AMPK–Adenosine Monophosphate-activated Protein Kinase; Aox3 – aldehyde oxidase 3; C/EBP–CCAAT-Enhancer-Binding Protein; CK–Compound K; Dct–dopachrome tautomerase; DIO–diet-induced obese; ERK–extracellular signal-regulated kinase; GLP-1 – Glucagon-Like Peptide-1; IGF-1 – Insulin-like Growth Factor 1; Nrf2 – nuclear factor erythroid 2-related factor 2; PGC–peroxisome proliferator-activated receptor gamma coactivator; PPAR–Peroxisome Proliferator-Activated Receptor; PI3K–phosphoinositide 3-kinase; PTP1B–Protein Tyrosine Phosphatase 1B; SREBP–Sterol Regulatory Element Binding Protein; TAG–Triacylglyceride; TCPTP–T Cell Protein Tyrosine Phosphatase; UCP1 – Uncoupling Protein 1; WAT–White Adipose Tissue.

## 2 Triterpenoids: a sustainable approach to promote healthy aging via metabolic restoration

### 2.1 Celastrol: a potent leptin sensitizer for restoring satiety signaling

Celastrol, a pentacyclic triterpene extracted from the roots of the *Tripterygium wilfordii* (thunder god vine) plant, has various pharmacological effects, including anti-inflammatory, anti-rheumatic, and anti-tumor activities (Lei et al., 2025). Moreover, it is currently considered one of the most potent natural compounds investigated for the pharmacological treatment of obesity (Liu et al., 2015; Zhao T et al., 2024).

Obesity is characterized by resistance or insensitivity to the hormone leptin (Zhao Y et al., 2024). Leptin, discovered in 1994 and encoded by the *obese* gene, is a circulating hormone primarily released from adipose tissue (Lu and Li, 2000; Wabitsch, 2022). Blood leptin levels rise when energy stores are replete. It binds to hypothalamic leptin receptors, which mediate a strong satiety signal (Wabitsch, 2022). Conversely, low physiological leptin signals energy deficiency, prompting the body to increase energy stores through food intake and minimize the energy required to maintain physiological functions. Therefore, leptin is commonly referred to as the ‘satiety hormone’ (Liu et al., 2015). When leptin resistance develops, the body cannot use leptin to regulate energy metabolism (Liu et al., 2015; Zhao T et al., 2024).

Research in mouse models identified celastrol as a leptin sensitizer. It showed that this triterpenoid can restore lost sensitivity by directly binding to and inhibiting the protein tyrosine phosphatases PTP1B and TCPTP in the hypothalamus, which inhibit the leptin signaling cascade. It results in a significant reduction in food intake and, thereby, body weight, while simultaneously restoring energy expenditure. Celastrol’s effectiveness depends on leptin signaling, as it was ineffective in leptin-deficient and leptin receptor-deficient mouse models–confirming its role as a true sensitizer rather than a nonspecific anorectic (Liu et al., 2015; Kyriakou et al., 2018; Zhao Y et al., 2024).

The potential of celastrol for combination therapies is supported by observations that, in celastrol-pretreated lean mice, exogenous leptin injection significantly reduced food consumption and body weight compared with leptin treatment alone. Thus, this observation demonstrates that increased leptin levels and enhanced sensitivity lead to maximal activation of the satiety signaling pathway (Liu et al., 2015).

Despite its therapeutic potential, the clinical application of celastrol remains challenging, in part, because of its narrow therapeutic window. In mouse models, high doses have been associated with increased blood pressure and reduced physical activity. Additionally, its poor formulation properties, such as low aqueous solubility and, in turn, limited bioavailability (i.e., the fraction of an orally administered drug that reaches the systemic circulation), and its instability under physiological conditions need to be carefully examined before large-scale clinical testing in humans is possible (Cascão et al., 2017; Saito et al., 2019; Shi et al., 2020; Staicu et al., 2024).

Celastrol tolerability depends highly on dose and treatment duration. Historical clinical trials have successfully treated inflammatory diseases such as rheumatoid arthritis and psoriasis, showing that celastrol can be effective and well tolerated within specific dosage ranges (Tao et al., 2002). However, the metabolic mechanisms underlying rheumatoid arthritis and psoriasis differ fundamentally from those of obesity. Therefore, these clinical trial outcomes cannot be translated to obese humans. Clinical evidence specifically relevant to obesity is still in its early stages. Currently, a phase 1 study (NCT05494112; clinical research, drug testing generally involves four consecutive study phases) is underway to evaluate the safety and tolerability of celastrol in healthy individuals, with the final results still being evaluated (Patel and Qvit, 2022).

To address limited plant material, the presence of other toxic compounds in the same plant tissue, and the lack of efficient chemical synthesis methods, Zhao *et al.* recently elucidated the complete biosynthetic pathway of celastrol in plants. This breakthrough enables its sustainable bioproduction via genetically modified *Saccharomyces cerevisiae*, ensuring reliable delivery for further research (Zhao et al., 2023).

### 2.2 Ursolic acid: enhancing energy expenditure via body recomposition

Ursolic acid (UA) is a pentacyclic triterpene carboxylic acid that occurs naturally in various leaves, fruits, and herbs, including apples, basil, peppermint, lavender, oregano, and thyme (Namdeo et al., 2023). As a bioactive, plant-derived metabolite, UA shows promising therapeutic potential for a wide range of pharmacological applications. Beyond modulating intra- and extracellular molecular pathways involved in apoptosis, metastasis, and inflammatory processes, UA and its derivatives exhibit significant potential in the prevention and treatment of diseases, such as malignancies, cardiovascular disease, and neurological disorders (Liu, 2005; Kashyap et al., 2016).

The most interesting aspect of UA is its potential for body recomposition. In a mouse model of diet-induced obesity, Kunkel *et al.* showed that UA administration promotes simultaneous growth of both skeletal muscle and brown adipose tissue (BAT) by enhancing the activation of the insulin-like growth factor 1 (IGF1)/kinase Akt (also known as protein kinase B) pathway (Kunkel et al., 2011).

In skeletal muscle, activated Akt stimulates muscle metabolism in two ways: it activates the mammalian target of rapamycin (mTOR) signaling pathway to promote protein synthesis and muscle hypertrophy, while inhibiting muscle atrophy processes mediated by FoxO transcription factors (Bodine et al., 2001; Kunkel et al., 2011; 2012; Schiaffino and Mammucari, 2011; Ogasawara et al., 2013; Katashima et al., 2017; Russell and Schneider, 2020).

In BAT, activated Akt signaling stimulates expression of key components necessary for brown adipocyte formation and preservation, including the peroxisome proliferator-activated receptor (PPARγ), the master regulator of adipogenesis; members of the CCAAT-enhancer-binding protein (C/EBP) family; and PR domain containing 16. As a main result, Uncoupling Protein 1 (UCP1) activity increases. Brown adipocytes feature a high mitochondrial content, which gives them their brown color. UCP1 is in the inner mitochondrial membrane. It uncouples mitochondrial respiration–that is, nutrient consumption–from adenosine triphosphate synthesis, thereby driving heat production without muscle contraction or shivering (non-shivering thermogenesis). Upregulation of UCP1 therefore, leads to a higher rate of fat burning and, consequently, enhanced weight loss (Kunkel et al., 2011; 2012; Brown and Scherer, 2023; Karanfil et al., 2026).

These findings show that UA effectively promotes the maintenance and growth of skeletal muscle mass and BAT. The associated increase in energy expenditure makes this triterpenoid a promising therapeutic agent for treating obesity (Kunkel et al., 2011; 2012; Katashima et al., 2017).

Despite these spectacular results in animal models, translating UA’s therapeutic efficacy into human clinical applications remains challenging. Recent meta-analyses indicate that UA supplementation at standard doses does not significantly reduce body weight (Lobo et al., 2021; Rafiee et al., 2024). UA’s poor oral bioavailability, a key factor in drug design and development, is the main reason for the lack of groundbreaking successes in humans (Staicu et al., 2024). The higher this fraction, the less drug is needed to reach the desired pharmacological effects. Several key parameters contribute to UA’s low absorption: poor solubility due to its high hydrophobicity, low membrane permeability, and rapid first-pass metabolism in hepatocytes and intestinal epithelium (Lee, 2020; Sun et al., 2020; Yu et al., 2020; Staicu et al., 2024). Researchers are currently exploring (in)organic structures and nanoparticles as delivery systems to overcome these pharmacokinetic limitations and improve absorption in humans, thereby achieving the tissue concentrations needed to activate the IGF1/Akt signaling pathway (Staicu et al., 2024).

### 2.3 Ginsenosides: modulating browning, adipogenesis, lipogenesis, and satiety signaling

Ginsenosides are a structurally heterogeneous group of triterpenoids mainly derived from plants of the genus *Panax* (Ginseng), Korean ginseng, and *Panax notoginseng* (Kim D et al., 2018; Zhang et al., 2021). They can be classified based on differences in their aglycone skeleton and the position and number of glycosyl groups into protopanaxadiols (PPD), including Rb1, Rg3, and Compound K (CK), and protopanaxatriols (PPT), including Rg1, Rg2, and Rh1. Current studies have demonstrated the strong potential of both primary parent ginsenosides (e.g., Rb1, Rg1, Rg3) and their bioactive metabolites, especially Compound K (CK), as potent anti-obesity agents due to their substantial ability to interfere with glucose and lipid metabolism (Huang et al., 2010; Oh and Chun, 2022; Wang et al., 2025). Crucially, the activity level and therefore the therapeutic potential of ginsenosides strongly depend on the degree of conversion: The biotransformation of major ginsenosides yields more active, deglycosylated counterparts (Wang et al., 2025).

The primary ginsenoside Rb1 exhibits potent anti-obesity effects, including the inhibition of lipogenesis. A key characteristic of Rb1 is its ability to activate browning processes in white adipose tissue (WAT) by stimulating the phosphorylation of adenosine monophosphate-activated protein kinase (AMPK) (Xiong et al., 2010; Zhou et al., 2019; Pu et al., 2022). Subsequently, the downstream expression of molecular markers such as UCP1 and master regulators such as PPARγ and peroxisome proliferator-activated receptor γ coactivator 1α is upregulated. *In vitro* and *in vivo* studies show that Rb1 increases the expression of UCP1 and master regulators, thereby promoting mitochondrial activity and biogenesis and, in turn, increasing energy expenditure via thermogenesis (Park H et al., 2019; Pu et al., 2022). Furthermore, this Rb1-mediated AMPK activation effectively stimulates cellular autophagy processes to degrade dysfunctional organelles and lipid droplets (Yuan et al., 2025). Additionally, it can improve insulin and leptin sensitivity, which is often impaired in obesity (Desjardins and Steinberg, 2018).

CK is an intestinal microbiota metabolite, mainly derived from the biotransformation of Rb1, and is characterized by higher bioavailability and solubility than its parent ginsenosides. It modulates several key molecular pathways, primarily resulting in (1) the inhibition of adipogenesis, (2) the attenuation of fatty acid synthesis, and (3) the reduction of oxidative stress (Oh and Chun, 2022).

By downregulating the expression of mRNAs involved in adipocyte differentiation, including PPARγ and C/EBPα, and suppressing the phosphorylation of extracellular signal-regulated kinase (ERK), p38, and Akt, this approach can significantly inhibit preadipocyte differentiation into mature adipocytes, thereby preventing the expansion of WAT without causing cytotoxic effects (Oh and Chun, 2022).

Additionally, CK activates the AMPK signaling pathway to inhibit key downstream transcriptional modulators of *de novo* synthesis of cytoplasmic fatty acids, such as acetyl-CoA carboxylase one and sterol regulatory element-binding protein 1c, thereby attenuating lipogenesis (Hardie et al., 2012; Jeon, 2016; Wang et al., 2018; Oh and Chun, 2022).

Obesity and diabetes are strongly associated with oxidative stress, characterized by elevated reactive oxygen species (ROS) levels (Huang et al., 2026). High nutrient intake increases oxidative stress and induces inflammation via the nuclear factor-κ-light-chain-enhancer of activated B cells (NF-κB) signaling pathways. Oxidative stress can result in lipid peroxidation, protein modification, and DNA damage (Li et al., 2023). CK modulates the balance between antioxidants and ROS by decreasing ROS production and increasing endogenous antioxidant levels (Forrester et al., 2018; Oh and Chun, 2022).

Another major ginsenoside, Rg1, also has anti-obesity effects by modulating both metabolism and energy intake. Among other effects, it inhibits lipogenesis and adipogenesis and promotes adipocyte browning by activating the AMPK pathway (Liu et al., 2018). Furthermore, Rg1 can significantly suppress pancreatic lipase activity, an enzyme that converts triglycerides into absorbable free fatty acids and glycerol. As a result, Rg1 inhibits triglyceride digestion, thereby reducing fat absorption and, consequently, lowering caloric uptake (Li and Ji, 2017). Beyond its digestive effects, Rg1 has recently been verified in rat models to significantly reduce hypothalamic oxidative stress signaling by promoting Nrf2 (nuclear factor erythroid 2-related factor 2)-related tyrosine metabolic pathways and downregulating key enzyme expression, such as Aldh3a1, Aox3, and Dct, thereby improving insulin and leptin resistance and restoring metabolic homeostasis (Huang et al., 2026).

Several animal studies verified the efficiency of the mechanisms described. In high-fat-diet-induced obese mice and rats, for example, treatment with Rg1 (often in combination with Rb1) effectively reduced body weight, blood lipids, body fat content, and total food intake (Li et al., 2018; Park S et al., 2019; Pu et al., 2022).

Rg3, however, was identified as the most potent in mediating appetite control. It was shown to stimulate glucagon-like peptide 1 (GLP-1) secretion, which, in turn, sends appetite-suppressing signals through the sympathetic nervous system, enhances satiety signaling, and thereby reduces food intake–all key aspects in obesity management (Kim et al., 2015).

Ginsenosides act not only at the cellular and enzymatic levels but also significantly improve obesity and its complications by modulating the composition of the gut microbiota (Zhuang et al., 2021). Many studies report that obesity development is associated with compositional and functional changes in the gut microbiota. The phyla *Firmicutes* and *Bacteroidetes* account for approximately 90% of the mammalian gut microbiota. Members of the *Firmicutes* phylum are highly involved in nutrient absorption and metabolism by synthesizing short-chain fatty acids, while *Bacteroidetes* species play a key role in immunomodulation (Cho, 2023). Early findings revealed a direct correlation between an enhanced F/B ratio and obesity in obese individuals. However, recent studies increasingly report an inverse or no relationship between these two factors, suggesting that presenting this ratio as a definitive biomarker for metabolic health is an oversimplification (Houtman et al., 2022). Indeed, the gut microbiota is modulated in obese individuals; however, the exact underlying molecular pathways require further elucidation (Zhuang et al., 2021).

While numerous studies document the success of ginsenoside treatment and its underlying molecular mechanisms in animal models and cell cultures, a breakthrough in human clinical trials to establish ginsenoside-based obesity treatment has yet to occur. Current human studies on ginsenosides show that translation remains a significant challenge. Only modest, often inconsistent reductions in body weight are observed (Miraghajani et al., 2020; Huang et al., 2025). A key limiting factor is the low oral bioavailability of ginsenosides in their native forms because of their large size, hydrophilic nature, and low cell membrane permeability. During metabolism, gut microbiota primarily convert parent ginsenosides into various bioactive secondary metabolites, such as CK, which exhibit significantly enhanced bioavailability but are often associated with rapid clearance (Wang et al., 2025). For this reason, gut microbiota profiles and individual genetic backgrounds play crucial roles in ginsenoside transformation (Kim, 2018).

To address these limitations, synthetic biology offers powerful technologies to advance the preclinical development of these compounds. Engineered probiotics offer a promising opportunity by enhancing gut colonization, modulating metabolic functions, and delivering targeted bioactive compounds (Duhan et al., 2025). By using genetically optimized microbes and their enzymes to biotransform primary ginsenosides *in situ*, the production of active metabolites can be decoupled from human microbiota variability (Son et al., 2024). However, while these tools can significantly improve production performance and structural bioavailability, they do not address critical translational factors for widespread clinical application, such as pharmacodynamic efficacy, long-term colonization dynamics in human trials and clinical efficacy, toxicity, regulatory hurdles, and long-term safety of genetically modified probiotics (Moon, 2024; Duhan et al., 2025).

As a complementary strategy, industrial *ex situ* production uses genetically optimized microorganisms to produce pure, bioactive ginsenosides at precisely defined dosages (Geraldi, 2020; Alenazy et al., 2022).

### 2.4 Oleanolic acid: promoting cellular longevity through telomerase activity

Oleanolic acid (OA), an isomer of UA, is a pentacyclic triterpenoid and a widespread secondary metabolite in plants, e.g., olive leaves and grape skins. It is known for its diverse pharmacological properties, including anti-inflammatory and antidiabetic activities (Castellano et al., 2022). In the context of obesity and its associated complications, oleanolic acid has shown beneficial effects *in vitro* and in animal models, mediated by complex, multi-targeted molecular mechanisms (Claro-Cala et al., 2022).

In animal models of obesity, OA showed anti-inflammatory effects. OA administration leads to overexpression of PPARα, predominantly in liver tissue, which, in turn, inhibits the expression of inflammation-related genes. Immune cells, primarily macrophages, play a key role during obesity-induced inflammatory processes (Shoelson et al., 2007). OA facilitates a macrophage phenotype switch from the highly pro-inflammatory M1 to the M2 phenotype, which secretes anti-inflammatory cytokines. As a result, levels of inflammatory factors such as interleukin-6 and tumor necrosis factor-α decrease (Ray et al., 2016; Claro-Cala et al., 2022; Zhang et al., 2024).

In addition to facilitating anti-inflammatory processes, OA modulates lipolysis and adipogenesis pathways by regulating key regulatory genes: The expression of C/EBPα and PPARγ, essential for adipocyte differentiation, is inhibited (Claro-Cala et al., 2022). Furthermore, OA suppresses key lipid-metabolism factors, such as C/EBPβ, PPARγ, and sterol regulatory element-binding protein 1c, thereby significantly reducing fat accumulation in liver tissue (Zhang et al., 2024).

Sun et al. (2024a) reported that OA mediates increased skeletal muscle mass by regulating the phosphoinositide 3-kinase/Akt signaling pathway, thereby inhibiting obesity-induced muscle atrophy and promoting protein synthesis.

Beyond these effects, OA acts as a potent agonist of the transmembrane G protein-coupled receptor 5 (TGR5), which is expressed in BAT. Activated TGR5 increases cellular energy production, thereby reducing fat tissue and body weight. In mice fed a high-fat diet, OA-mediated TGR5 activation improves mitochondrial biogenesis, thereby suppressing increases in fat mass and body weight (Ono et al., 2011; Mikami et al., 2021). Furthermore, TGR5 is expressed in enteroendocrine L cells, where OA activation induces GLP-1 secretion. As described for ginsenoside Rg3, this release improves, among other things, insulin sensitivity and, in turn, appetite regulation (Ono et al., 2011).

Similar to Rg1, which reduces fat absorption via lipase suppression in the pancreas, OA effectively reduces glucose absorption–and thereby calorie uptake–from the intestine by inhibiting carbohydrate-digesting enzyme activity, especially of α-glucosidase, through enzyme binding (Li and Ji, 2017; Ding et al., 2018; Xie et al., 2021).

Another potent effect of OA worth mentioning is its ability to enhance telomerase activity, an enzyme that can reverse age-related telomere shortening. Because telomere length decreases with age and is a key biomarker of somatic cell aging, when it falls below a certain threshold, cell growth is restricted, and cells undergo cellular senescence or apoptosis. In this context, OA may modulate cellular aging pathways. *In vitro* studies with human peripheral blood mononuclear cells show that treatment with 1 μg/mL of OA for 24–72 h can enhance telomerase activity by up to 5.9-fold, potentially slowing obesity-induced accelerated cellular senescence and supporting cellular healthspan. However, these findings are limited to the *in vitro* system described, and further *in vivo* and human clinical studies are needed to investigate OA’s mode of action (Tsoukalas et al., 2019).

### 2.5 Betulinic acid: decreasing fat accumulation independently of food intake

Betulinic acid (BA), a pentacyclic triterpenoid, occurs naturally in numerous plant species and can be chemically or enzymatically synthesized from its precursor, betulin. It exhibits, among others, anti-inflammatory, antioxidant, and anti-cancer properties by modulating fundamental metabolic pathways, including AMPK and NF-κB signaling. In treating obesity and its associated disorders, BA can improve insulin sensitivity, reduce hepatic steatosis (≥5% of hepatocytes), and suppress inflammatory responses (de Melo et al., 2009; Kim et al., 2012; Csuk, 2014; Azevedo et al., 2025). Regarding its anti-inflammatory mechanism, BA modulates ERK, Akt, and NF-κB signaling pathways. Furthermore, it inhibits phospholipase A2 activity and nitric oxide production, which act as inflammatory mediators in the progression of certain inflammatory diseases (Viji et al., 2011; Azevedo et al., 2025).

*In vivo* studies have shown that mice on a high-fat diet treated with BA exhibited significantly decreased body weight, tissue adiposity (BAT and WAT), and lipid droplet size, as well as blood glucose, plasma triacylglycerol (TAG), and cholesterol levels compared to controls not fed BA. Notably, these effects occurred independently of food intake (de Melo et al., 2009; Kim et al., 2019). Various molecular mechanisms of action underlie these effects: drastically increased AMPK activity and therefore phosphorylation, which directly triggers autophagy processes while simultaneously resulting in decreased levels of mRNA encoding key enzymes in TAG synthesis and reduced gene and protein expression of transcription factors mediating adipogenesis (PPARγ) (Al-Mutary et al., 2019; Kim et al., 2019). Furthermore, BA exhibits TGR5 agonist activity like OA, increasing energy expenditure by promoting mitochondrial biogenesis (Genet et al., 2010; Ono et al., 2011).

In rat models, BA has been shown to promote weight loss and decrease abdominal fat accumulation. These effects likely result from direct inhibition of pancreatic lipase activity, thereby suppressing an increase in plasma TAG levels, potentially by inhibiting intestinal lipid absorption. Moreover, BA showed a strong lipolytic effect, potentially accelerating the digestion of adipose tissue lipids into glycerol and free fatty acids (de Melo et al., 2009; Kim et al., 2012; Zhu et al., 2024).

In summary, preclinical studies have demonstrated the significant therapeutic potential of BA for managing obesity and obesity-related disorders. However, clinical trials are still in early phases, focusing on establishing efficacy, safety profiles, and optimal protocols for human use (Zhu et al., 2024; Azevedo et al., 2025).

### 2.6 Additional triterpenoids with promising therapeutic potential

In addition to the five triterpenoids mentioned above, several others show promising anti-obesity potential by modulating metabolic and inflammatory pathways.

- Boswellic Acids:

Boswellic Acids, a group of pentacyclic triterpenoids derived from the oleo-gum resin of *Boswellia serrata* trees, exhibit potent anti-inflammatory and antioxidant properties. These compounds modulate key signaling pathways for meta-inflammation by inhibiting the NF-κB signaling cascade, suppressing cyclooxygenase-2 and lipoxygenase enzymes–primarily 5-lipoxygenase –, and subsequently downregulating the secretion of proinflammatory cytokines, such as interleukin-6 and tumor necrosis factor-α (Abdelnour et al., 2025; Kuwar and Kalia, 2025; Mahto et al., 2025).

Beyond their traditional anti-inflammatory role, several studies have demonstrated the benefits of boswellic acids and *Boswellia* extracts in maintaining or restoring metabolic homeostasis: In animal models, the water-soluble fraction of *B. serrata* exhibits anti-obesity and hypolipidemic effects by lowering the levels of total cholesterol, TAGs, free fatty acids, and low-density lipoproteins (Mahdian et al., 2020). At the compound level, an *in vitro* study reported that 3-O-acetyl-11-keto-β-boswellic acid directly induces lipolysis in mature human adipocytes (Liu et al., 2013; Mahdian et al., 2020).

Despite these effects, clinical application is currently hindered by significant challenges, including inconsistent extraction processes, low oral bioavailability (due to poor aqueous solubility and rapid first-pass metabolism), and a lack of large-scale human clinical trials evaluating purified single compounds to establish well-defined safety and dosing protocols (Mahto et al., 2025).

- Mogrosides:

Mogrosides are triterpenoids predominantly sourced from monk fruit (*Siraitia grosvenorii*). In recent years, mogrosides, especially mogroside V (MOG-V), have become known as high-intensity, non-caloric sweeteners. MOG-V is about 250-300 times as sweet as sucrose and has only 1/50th the calories (Shivani et al., 2021; Zhang et al., 2025). While recognized as GRAS (Generally Recognized as Safe) by the U.S. Food and Drug Administration and approved as safe in various countries (e.g., Japan, China, Canada, Australia), mogrosides remain only partially approved in the EU due to gaps in toxicological data (Mooradian et al., 2017; Kaim et al., 2025; Wazir et al., 2025). Beyond their use as sugar substitutes to reduce calorie intake, both mogroside-rich extracts and purified components like MOG-V exhibit direct anti-obesity effects by activating the AMPK signaling pathway. This activation regulates protein and gene expression related to lipid metabolism, thereby inhibiting *de novo* lipogenesis, enhancing fatty acid oxidation, and promoting lipid breakdown in preclinical models (Zhang et al., 2018; Li et al., 2020; Chen et al., 2024). Further investigation of the specific metabolic effects of individual mogrosides is needed.

- Cucurbitacins:

Cucurbitacins are tetracyclic triterpenoids found in the Cucurbitaceae family. They are classified into 12 classes, including cucurbitacin B and E (CuE), the most prominent representatives in terms of metabolic impact (Nie et al., 2024). Cucurbitacins have strong pharmacological potential due to their bioactivity, including anti-inflammatory, anti-tumor, anti-diabetic, and anti-obesity activities (Kaushik et al., 2015; Tuli et al., 2023; Ahamad et al., 2026).

In mouse models, CuE was verified to reduce adipogenesis in adipocytes. CuE administration decreased adipocyte hypertrophy, visceral obesity, and lipogenic gene expression by inhibiting the Janus kinase/signal transducer and activator of transcription 5 (JAK-STAT5) signaling pathway in visceral fat tissue, thereby reducing PPARγ expression. Furthermore, CuE has been shown to activate AMPK in mouse muscle tissue, leading to enhanced glucose uptake and the reversal of high-fat diet-induced insulin resistance, thereby restoring metabolic homeostasis (Murtaza et al., 2017).

Another derivative, cucurbitacin B, can stimulate GLP-1 secretion in enteroendocrine L-cells via AMPK activation, thereby potentially increasing satiety (Kim et al., 2015; Kim K et al., 2018).

Despite the evident therapeutic potential of cucurbitacins, the main issue with their clinical application for long-term management of chronic obesity is their inconsistent, highly dose-dependent toxicity, which makes identifying an optimal therapeutic window extremely challenging (Ahamad et al., 2026).

- Asiaticosides & Madecassosides:

Asiaticosides and Madecassosides are pentacyclic triterpene saponins derived from *C. asiatica*, a herb used in traditional medicine, e.g., to increase brain function, lower blood sugar levels, or improve blood circulation. Besides asiatic and madecassic acids, they mostly contribute to the therapeutic potential of *Centella asiatica*, including wound-healing, anti-inflammatory, and anticancer effects (Tripathy et al., 2022; Hong et al., 2023; Barinda et al., 2025).

Recent studies have shown that the suspension of powdered *C. asiatica* leaves exhibits anti-obesity effects in mouse models by modulating metabolic pathways. This plant suspension suppresses lipid levels and enhances sensitivity to leptin and insulin *in vivo* (Chang et al., 2023). Regarding the underlying mechanisms, *C. asiatica* extract inhibits preadipocyte differentiation by reducing the expression of adipogenesis-related genes (PPARγ, C/EBPα). It suppresses the activity of key lipogenic enzymes (fatty acid synthase, stearoyl-CoA desaturase 1). It also inhibits pancreatic lipase activity, thereby reducing intestinal fatty acid absorption and lipid accumulation (Hong et al., 2023). Despite these promising results, further research is needed to determine the extent to which individual bioactive components of the *C. asiatica* extract contribute to the observed anti-obesity effects.

Besides affecting metabolic levels, triterpenoids in *C. asiatica* extract can promote collagen synthesis by remodeling the collagen matrix and provide neuroprotection, making them potent factors for preserving healthspan (Bandopadhyay et al., 2023; Thong-On et al., 2024).

## 3 Discussion

With obesity classified by the World Health Organization as one of the most serious global epidemics of our time, treating this disease and its associated severe disorders must be a top priority to preserve human healthspan. This review highlights the strong potential of natural triterpenoids for promoting healthy aging. Nevertheless, a critical assessment of clinical translation readiness shows that the highlighted triterpenoids are at different stages on the path to clinical application, and their key bottlenecks must be addressed before they can be used effectively as therapeutic agents. While primary compounds like celastrol and cucurbitacins show substantial molecular and cellular efficacy, their use is severely limited by narrow therapeutic windows and dose-dependent off-target toxicities, leading to gastrointestinal, hepatotoxic, and even neurotoxic effects (Yuan Sun et al., 2024; Ahamad et al., 2026). In contrast, triterpenoids such as ursolic acid, the ginsenoside family, and oleanolic acid possess substantially wider safety margins and are well-tolerated at functional doses (Woźniak et al., 2015; Park S et al., 2019; Pu et al., 2022). However, they are highly limited by their extreme hydrophobicity, low membrane permeability, and rapid systemic clearance, resulting in low bioavailability and, in the case of ginsenosides, obligatory dependence on microbiota biotransformation. Additionally, they have significant drug-drug interaction potential. Among others, they can modulate the activity of several cytochrome P450 (CYP) enzymes, such as CYP1A2 and CYP3A4, interfering with co-administered drugs metabolized primarily by CYPs (Kim et al., 2004).

Consequently, these compound-specific pharmacokinetic and safety profiles, as well as additional limitations, including inconsistencies in animal models, variable chemical compositions of triterpenoid preparations, and a lack of standardized dosing protocols, must be systematically optimized. Moreover, long-term safety data and human clinical trials are extremely limited. Therefore, the scope must expand from conventional approaches towards advanced synthetic biology strategies, such as innovative delivery systems (Table 2), engineered probiotics, or the production of triterpenoids using genetically modified microorganisms, to close the gap between laboratory success and clinical application. Importantly, these innovative strategies also face regulatory and technical hurdles. Namely, nanotechnology-based delivery systems face limitations, such as nanomaterial toxicity, immunogenicity, large-scale manufacturing difficulties, and regulatory barriers (Babu and Ravi, 2026); engineered probiotics are constrained by factors such as human and environmental safety concerns, genetic stability during clinical application, and clinical and experimental challenges like heterogeneity, harmful effects, and high costs (Singh et al., 2025). The use of triterpenoid-producing engineered microorganisms is limited by scale-up challenges, including genetic instability and varying production parameters (Abbate et al., 2023).

**TABLE 2:** Nanotechnology-based drug delivery systems for improving pharmacokinetics and bioavailability of the selected primary triterpenoids.

Triterpenoid	Strategy & model	Key pharmacokinetic and functional outcomes	REFs
Celastrol	PEG-PCL nanoparticles DIO C57BL/6 mice	5 or 7.5 mg/kg/d for 3 weeks 4.6-fold increased AUC_0-t_ 7.5-fold increased c_max_; equally effective in reducing body weight and fat accumulation; less gastrointestinal toxicity than free celastrol via suppression of colonic and gastrointestinal inflammation; improved insulin resistance	(Zhao et al., 2019)
Celastrol	MMP-2-sensitive dextran sulfate with PVGLIG peptide linkage nanoparticle micelles; DIO C57 mice)	PVGLIG cleavage by MMP-2 enzymes triggers celastrol release at inflammatory sites; effective weight loss with minimized systemic toxicity and insulin resistance; restoration of homeostasis of blood glucose level	(Chen et al., 2022; Chan et al., 2024)
Celastrol	BSA nanoparticles; DIO mice	significantly improved cellular uptake; outperforms free celastrol in reducing lipid accumulation, improving insulin sensitivity, and driving mitigation of inflammation	(Fan et al., 2022)
Ursolic acid	ASDs via Soluplus/TPGS/choline combination; human colorectal adenocarcinoma cell line (Caco-2) cells; DIO Sprague-Dawley rats	oral bioavailability of ASD is 19 times higher than that of crude UA; superior dissolution properties, moderate intestinal membrane permeability, and a significantly reduced efflux rate	(Zhao T et al., 2024)
Oleanolic acid	SNEDDS; DIO Sprague-Dawley rats	significantly improved dissolution and 2.4-fold increased oral bioavailability vs. crude suspension	(Xi et al., 2009)
Betulinic acid	cyclodextrin-based nanocarrier; Caco-2 cells, Sprague-Dawley rats	enhanced dissolution and permeability, 7.41-fold enhanced plasma AUC levels compared to native drug (100 mg/kg, 48 h)	(Godugu et al., 2014; Saneja et al., 2018)

Abbreviations: ASD–amorphous solid dispersions; AUC–area under the curve; BSA–bovine serum albumin; DIO–diet-induced obese; MMP2 – metalloproteinase-2; mPEG–monomethoxy polyethylene glycol; PCL–poly (ε-caprolactone); PEG–polyethylene glycol; PLGA–polylactic-co**-**glycolic acid; SNEDDS–self-nano-emulsified drug delivery system; TPGS–triphenyl phosphonium-tocopherol polyethylene glycol succinate; UA–ursolic acid.

Only by systematically addressing these barriers can these advanced biotechnological approaches pave the way for a complementary strategy. Despite their promising potential as future therapeutics, the clinical use of natural triterpenoids warrants a direct comparison of their efficacy with established pharmacotherapies. In clinical trials, treatment with semaglutide and tirzepatide achieved average weight loss of approximately 15% and 20%, respectively (Bergmann et al., 2023; Aronne et al., 2025). In contrast, translating the potent results of triterpenoids from animal models and cell lines into human clinical applications remains challenging, as reflected in no significant or only modest, often inconsistent reductions in human body weight. Therefore, the full potential of triterpenoids will be realized primarily by viewing them not as stand-alone competitors to standard pharmacotherapies, but as adjunctive, complementary components. Their clinical value lies in their versatile spectrum of multi-target mechanisms that support metabolic health through long-term restoration, alongside established treatments.
